# Simplifying prediction of disease progression in pre-symptomatic type 1 diabetes using a single blood sample

**DOI:** 10.1007/s00125-021-05523-2

**Published:** 2021-08-02

**Authors:** Naiara G. Bediaga, Connie S. N. Li-Wai-Suen, Michael J. Haller, Stephen E. Gitelman, Carmella Evans-Molina, Peter A. Gottlieb, Markus Hippich, Anette-Gabriele Ziegler, Ake Lernmark, Linda A. DiMeglio, Diane K. Wherrett, Peter G. Colman, Leonard C. Harrison, John M. Wentworth

**Affiliations:** 1 Department of Population Health and Immunity, Walter and Eliza Hall Institute, Parkville, VIC Australia; 2 Department of Medical Biology, University of Melbourne, Parkville, VIC Australia; 3 Department of Bioinformatics, Walter and Eliza Hall Institute, Parkville, VIC Australia; 4 University of Florida Diabetes Institute, Gainesville, FL USA; 5 Department of Pediatrics and Diabetes Center, University of California at San Francisco, San Francisco, CA USA; 6 Center for Diabetes and Metabolic Diseases, Indiana University School of Medicine, Indianapolis, IN USA; 7 Barbara Davis Center, University of Colorado School of Medicine, Aurora, CO USA; 8 Helmholtz Zentrum München, Institute of Diabetes Research, German Research Center for Environmental Health, Munich-Neuherberg, Germany; 9 Forschergruppe Diabetes, Technical University Munich at Klinikum rechts der Isar, Munich, Germany; 10 Department of Clinical Sciences, Lund University/CRC, Skåne University Hospital, Malmö, Sweden; 11 Division of Pediatric Endocrinology, Department of Pediatrics, Indiana University School of Medicine, Indianapolis, IN USA; 12 Division of Endocrinology, Department of Pediatrics, Hospital for Sick Children, University of Toronto, Toronto, ON Canada; 13 Department of Diabetes and Endocrinology, Royal Melbourne Hospital, Parkville, VIC Australia

**Keywords:** Disease progression, OGTT, Prediction, Prevention, Risk stratification, Type 1 diabetes

## Abstract

**Aims/hypothesis:**

Accurate prediction of disease progression in individuals with pre-symptomatic type 1 diabetes has potential to prevent ketoacidosis and accelerate development of disease-modifying therapies. Current tools for predicting risk require multiple blood samples taken during an OGTT. Our aim was to develop and validate a simpler tool based on a single blood draw.

**Methods:**

Models to predict disease progression using a single OGTT time point (0, 30, 60, 90 or 120 min) were developed using TrialNet data collected from relatives with type 1 diabetes and validated in independent populations at high genetic risk of type 1 diabetes (TrialNet, Diabetes Prevention Trial–Type 1, The Environmental Determinants of Diabetes in the Young [1]) and in a general population of Bavarian children who participated in Fr1da.

**Results:**

Cox proportional hazards models combining plasma glucose, C-peptide, sex, age, BMI, HbA_1c_ and insulinoma antigen-2 autoantibody status predicted disease progression in all populations. In TrialNet, the AUC for receiver operating characteristic curves for models named M_60_, M_90_ and M_120_, based on sampling at 60, 90 and 120 min, was 0.760, 0.761 and 0.745, respectively. These were not significantly different from the AUC of 0.760 for the gold standard Diabetes Prevention Trial Risk Score, which requires five OGTT blood samples. In TEDDY, where only 120 min blood sampling had been performed, the M_120_ AUC was 0.865. In Fr1da, the M_120_ AUC of 0.742 was significantly greater than the M_60_ AUC of 0.615.

**Conclusions/interpretation:**

Prediction models based on a single OGTT blood draw accurately predict disease progression from stage 1 or 2 to stage 3 type 1 diabetes. The operational simplicity of M_120_, its validity across different at-risk populations and the requirement for 120 min sampling to stage type 1 diabetes suggest M_120_ could be readily applied to decrease the cost and complexity of risk stratification.

**Graphical abstract:**

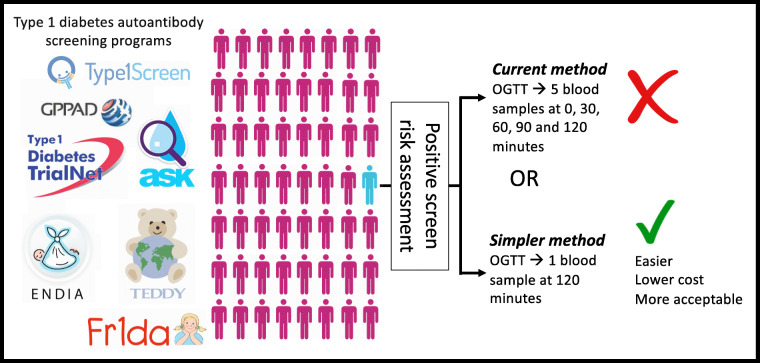

**Supplementary Information:**

The online version of this article (10.1007/s00125-021-05523-2) contains peer-reviewed but unedited supplementary material.

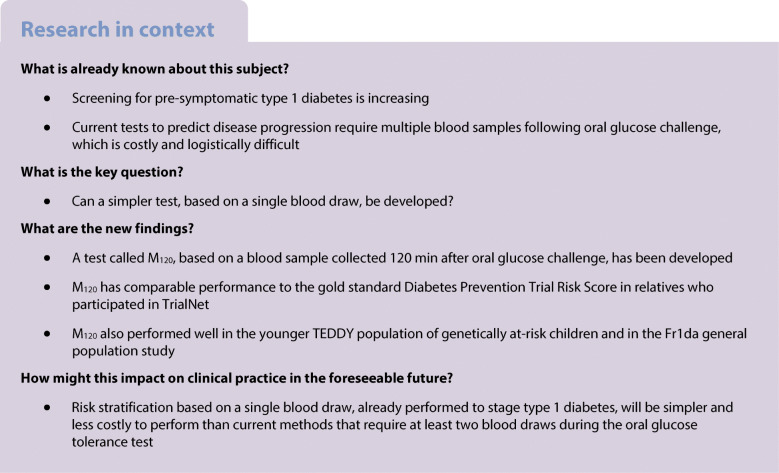



## Introduction

Interest in autoantibody screening for type 1 diabetes risk has increased following the demonstration that early diagnosis prevents ketoacidosis [1–3] and provides opportunities to delay disease progression with immune therapies [4, 5]. Type 1 diabetes screening programmes underway in Europe, North America, Australia and New Zealand test for autoantibodies against insulin (IAA), GAD (GADA), insulinoma antigen-2 (IA-2A) and zinc transporter-8 (ZnT8A) [6–8]. The presence of two or more autoantibodies confers a very high lifetime risk of disease progression to insulin dependence [9] and has prompted the reclassification of type 1 diabetes as an autoimmune beta cell disorder defined primarily by immune rather than metabolic markers [10, 11].

Type 1 diabetes is now diagnosed when two or more islet autoantibodies are detected. Three disease stages based on oral glucose tolerance and HbA_1c_ have been defined. Stage 1 is defined by normal glucose tolerance and HbA_1c_, and stage 2 by impaired glucose tolerance and HbA_1c_ from 39 to 46 mmol/mol (5.7% to 6.4%), inclusive [11]. Both of these stages are asymptomatic. Stage 3 satisfies current diagnostic criteria for diabetes mellitus [12] and is usually accompanied by symptoms of hyperglycaemia. While this staging system is important for helping the medical and lay communities understand the progression of a largely silent autoimmune disease, staging is also used to determine eligibility for prevention trials. To differentiate between type 1 diabetes stages 1, 2 and 3, autoantibody-positive individuals undergo OGTTs, in which glucose is measured at baseline and 120 min after the glucose load. OGTTs performed in a number of research studies have collected glucose values at the 30, 60 and 90 min time points to further define risk characteristics [13]. However, these additional time points greatly increase the costs and complicate the logistics of the OGTT. In order to conserve limited resources and increase participation, some screening programmes are transitioning from a multiple-time-point OGTT to the standard clinical model in which sampling is performed only at baseline and 120 min.

The rate of progression from early-stage (1 or 2) type 1 diabetes to stage 3 type 1 diabetes is highly relevant for affected individuals and a critical determinant of both the sample size and duration of prevention trials. Younger age of seroconversion and the presence of IA-2A are associated with an increased risk of disease progression and have been used to predict its rate [14, 15]. However, more accurate risk stratification is afforded by considering glucose and C-peptide excursions during the OGTT. Currently, three stratification tools for predicting disease progression in pre-symptomatic type 1 diabetes have been validated. The Diabetes Prevention Trial Risk Score (DPTRS) incorporates age, BMI, and glucose and C-peptide at five OGTT time points [16], whereas the simpler Index60 endpoint is based on fasting C-peptide and the glucose and C-peptide at the 60 min time point [17]. The recently reported DPTRS60 score combines age and BMI with the inputs used to calculate Index60 [18]. These tools were developed using first-degree relative data from the Diabetes Prevention Trial–Type 1 (DPT-1) that began over 25 years ago, and performed initial participant screening with older assays for islet autoantibodies and C-peptide. These models were trained using data from single- as well as multiple-autoantibody-positive individuals, potentially limiting their applicability to the current staging paradigm that requires the presence of two or more autoantibodies [11]. In addition, the inputs used to develop DPTRS and Index60 were selected on the basis of *univariate* association with the outcome of type 1 diabetes, potentially missing interactions that contribute to disease risk.

Screening for type 1 diabetes autoantibodies, now extending into the general population [19], would benefit practically from the development of a broadly applicable and simple to administer tool to assess risk of disease progression from stage 1 or 2 to stage 3 type 1 diabetes, based on fewer OGTT time points. Our recent success in validating a model based on single-time-point measures to estimate beta cell function in stage 3 disease [20] led us to hypothesise that a simpler tool could be devised to assess risk of progression to stage 3. In the present study, we used the large Type 1 Diabetes TrialNet dataset [6] to develop and validate models based on a single OGTT time point that accurately predicted progression from stage 1 or 2 to stage 3 type 1 diabetes in other at-risk populations.

## Methods

### Data collection

Each protocol was approved by a human research ethics committee and was carried out in accordance with the Declaration of Helsinki as revised in 2008.

The TrialNet TN-01 Pathway to Prevention Study (NCT00097292) is an islet autoantibody screening and metabolic monitoring programme that has operated in North America, Europe, Australia and New Zealand since 2004. Individuals aged up to 45 years with a first-degree relative with type 1 diabetes and those aged up to 20 years with a first/second/third-degree relative with type 1 diabetes are screened for IAA, GADA, IA-2A and ZnT8A. TN-01 data current to December 2019 were downloaded in January 2020 and erroneous outlier values removed. Eligibility required fasting glucose <7.0 mmol/l (126 mg/dl), 120 min glucose <11.1 mmol/l (200 mg/dl), HbA_1c_ <48 mmol/mol (6.5%), age at least 2 years and BMI between 12 and 40 kg/m^2^. To enter the TrialNet multiple-autoantibody training population, participants needed complete data for the input measures of interest (electronic supplementary material [ESM] Table 1) and had an OGTT either at the same time that they first tested positive to multiple autoantibodies or at their next study visit (median [Q1, Q3] time between screening and OGTT 1.8 [0.3, 3.0] months). The TrialNet multiple-autoantibody validation population comprised individuals who met the same glucose, HbA_1c_, age and BMI criteria, and who had all measures required to calculate the DPTRS and the newer risk scores. These participants were not included in the training population because they underwent OGTT testing two or more visits after screening positive to multiple autoantibodies, lacked data for HLA genotype or did not have data for ZnT8A, which was only introduced into TrialNet in 2012. The median [Q1, Q3] time between screening and OGTT in the training population was 3.2 [1.5, 9.8] months. The TrialNet single-antibody population comprised 612 participants who tested positive to only one autoantibody and who underwent an OGTT that returned normal or impaired glucose tolerance and HbA_1c_ <48 mmol/mol (6.5%).

Data for the DPT-1 (NCT00004984) and The Environmental Determinants of Diabetes in the Young (TEDDY) study (NCT00279318) were obtained from the National Institute of Diabetes and Digestive and Kidney Diseases data repository in March and April 2020. DPT-1 recruited relatives with stage 1 or 2 type 1 diabetes between 1994 and 2003 and showed that neither parenteral nor oral insulin delayed progression to stage 3 [21, 22]. DPT-1 participants were positive for islet cell antibodies by indirect immunofluorescence assay and negative for the protective HLA-DQA1*01:02-DQB1*06:02 haplotype. Some assays for IAA were performed during the study whereas other IAA measurements, and all GADA and IA-2A measurements, were performed retrospectively on stored samples. TEDDY is a birth cohort study that enrolled 8668 North American and European newborns whose HLA genotype or family history conferred an increased risk of type 1 diabetes [23]. Data for multiple-autoantibody-positive children who had undergone a limited OGTT (blood sampling at 120 min) were extracted. For both DPT-1 and TEDDY, participants who had 120 min glucose of 11.1 mmol/l (200 mg/dl) or more, those who were missing data needed to calculate risk scores and those who had not been followed beyond their first OGTT were excluded.

The Fr1da study (NCT04039945) enrolled children aged 2 to 6 years from the general Bavarian population [7]. Children who screened positive for two or more islet autoantibodies were invited to undergo an OGTT with blood sampling at 0, 30, 60, 90 and 120 min. Participants with missing results for BMI, HbA_1c_ and IA-2A were excluded. Data were current to March 2020.

Stage 2 type 1 diabetes was defined as a fasting glucose of 5.6 to 7.0 mmol/l (100 to 125 mg/dl), a glucose at 30–90 min greater than 11.1 mol/l (200 mg/dl), a 120 min glucose of 7.8 to 11.1 mmol/l (140 to 199 mg/dl) and/or HbA_1c_ of 39 to 46 mmol/mol (5.7% to 6.4%), inclusive [11]. Stage 3 type 1 diabetes was defined using ADA criteria for diabetes mellitus [12]. The dose of glucose used in OGTTs was 1.75 g/kg to a maximum of 75 g. C-peptide was measured by radioimmunoassay in DPT-1 and for other studies using the TOSOH autoanalyser (TOSOH, South San Francisco, CA, USA). In TrialNet, DPT-1 and TEDDY, HbA_1c_ was measured using ion-exchange high-performance liquid chromatography on TOSOH autoanalysers and standardised using the Diabetes Control and Complications Trial reference method. HbA_1c_ measurements for Fr1da were performed at the participant’s local clinical laboratory.

### Analyses

The *glmulti* (v1.0.8) [24] and *survival* (v3.1-12) [25] packages of R software (v3.6.3; www.r-project.org) were used to build all possible single OGTT time point Cox proportional hazards regression models to predict progression from stage 1/2 to stage 3 type 1 diabetes using all possible combinations of the inputs listed in ESM Table 1. Models were then ranked by their Akaike’s information criterion (AIC) score. For each OGTT time point, the simplest model that was within 2 AIC units of the model with the lowest AIC score was selected for further testing. Coefficients for these models, named M_0_, M_30_, M_60_, M_90_ and M_120_, are presented in ESM Table 2. Model calibration testing was performed with the Greenwood–D’Agostino–Nam test using the GND.calib R function [26], where deciles with few events were integrated into the next decile, as appropriate, and *p* > 0.05 considered no evidence of poor fit.

Equations for the DPTRS, DPTRS60, Index60 and M_120_ risk tools are provided below, where the units for BMI, age, glucose, C-peptide and HbA_1c_ are, respectively, kg/m^2^, years, mg/dl, ng/ml and percentage units. Sex was assigned a score of 1 for male and 2 for female, and IA-2A status assigned 0 for absent and 1 for present. Glucose is converted from mmol/l to mg/dl by multiplying by 18; C-peptide is converted from nmol/l to ng/ml by dividing by 3.00; and HbA_1c_ is converted from mmol/mol to percentage units by adding 23.5 and then dividing by 10.93.

DPTRS = 1.569×log_e_(BMI) − 0.056×(age) + 0.00813×(sum of glucose from 30 to 120 min) − 0.0848×(sum of C-peptide from 30 to 120 min) + 0.476×log_e_(fasting C-peptide) [16]

DPTRS60 = 1.364×log_e_(BMI) − 0.065×(age) + 0.465×log_e_(fasting C-peptide) + 0.019×(60 min glucose) − 0.311×(60 min C-peptide) [18]

Index60 = 0.3695×log_e_(fasting C-peptide) + 0.0165×(60 min glucose) − 0.3644×(60 min C-peptide) [17]

M_120_ = 0.448×(sex) + 0.631×(IA-2A) − 0.0302×(age) + 0.0605×(BMI) + 1.380×(HbA_1c_) + 0.0265×(120 min glucose) − 0.191×(120 min C-peptide)

Prism software (v8.3.1g for Mac; GraphPad, San Diego, CA, USA) was used to perform Mann–Whitney tests for inter-group comparisons, to chart Kaplan–Meier survival curves of groups above and below the median value, and to compare the curves using the logrank (Mantel–Cox) test. AUC analysis of receiver operating characteristic (ROC) plots and comparisons of different prediction models were performed using the *pROC* package in R [27]. Calculations for sensitivity (*TP*/[*TP* + *FN*]), specificity (*TN*/[*TN* + *FP*]) and accuracy ([*TP* + *TN*]/*N*) used the median value as the risk threshold, where *TP*, *TN*, *FP*, *FN* and *N* are true-positives, true-negatives, false-positives, false-negatives and total number of participants, respectively.

### Statement of informed consent

Informed consent was obtained from all individual participants and, for children, their parents or legal guardians.

## Results

Models to predict risk of progression from stage 1 or 2 to stage 3 type 1 diabetes were developed using data from 1208 TrialNet participants who screened positive to at least two of IAA, GADA, IA-2A and ZnT8A and underwent an OGTT at the same time or at their next study visit. The median [Q1, Q3] age of this ‘training’ population was 9.3 [6.2, 13.3] years and 56% were male (Table 1). Five models, termed M_0_, M_30_, M_60_, M_90_ and M_120_, were developed using glucose and C-peptide measures obtained, respectively, at the 0, 30, 60, 90 and 120 min time points of the OGTT (ESM Table 2). These models also included age, sex, HbA_1c_ and IA-2A status, and all but M_0_ included BMI. Their performance characteristics in the TrialNet training population, together with those of DPTRS, DPTRS60 and Index60, are presented in Table 2.

**Table 1:** Population characteristics

Characteristic	TrialNet training dataset (*N*=1208)	TrialNet validation dataset (*N*=864)	TrialNet single-antibody dataset (*N*=612)	DPT-1 dataset (*N*=601)	TEDDY dataset (*N*=209)	Fr1da dataset (*N*=80)
Age (years)	9.3 [6.2, 13.3]; 2.0–55.3	9.9 [6.2, 14.5]; 2.1–49.7	13.0 [8.6, 29.9]; 2.2–49.5	11.2 [8.3, 15.5]; 2.9–46.0	6.6 [5.3, 7.9]; 2.9–10.7	4.3 [3.3, 5.5]; 2.1–6.7
Male, %	56	51	48	55	61	49
Years followed after 1st OGTT	1.8 [0.8, 3.2]; 0.1–12.0	2.4 [1.0, 5.0]; 0.04–14.6	2.2 [1.1, 4.0]; 0.2–11.5	3.1 [1.8, 4.6]; 0.1–7.2	2.1 [1.3, 2.4]; 0.1–5.3	3.1 [2.0, 4.0]; 0.4–4.9
Reaching stage 3 T1D, %	20	39	5.4	35	16	11
BMI (kg/m^2^)	17.5 [15.7, 21.3]; 12.0–39.4	18.5 [16.1, 23.1]; 12.5–39.8	20.6 [16.8, 25.6]; 12.8–39.8	18.8 [16.3, 22.4]; 12.5–38.1	15.8 [14.9, 17.2]; 12.7–26.0	15.6 [14.7, 16.3]; 12.9–20.7
HbA_1c_ (mmol/mol)	32 [30, 34]; 18–46	32 [30, 34]; 20–46	32 [30, 34]; 19–45	36 [32, 38]; 18–46	33 [32, 36]; 27–43	33 [31, 37]; 25–40
HbA_1c_ (%)	5.1 [4.9, 5.3]; 3.8–6.4	5.1 [4.9, 5.3]; 4.0–6.4	5.1 [4.9, 5.3]; 3.9–6.3	5.4 [5.1, 5.6]; 3.8–6.4	5.2 [5.1, 5.4]; 4.6–6.2	5.2 [5.0, 5.5]; 4.4–5.8
HLA^a^						
*DR3*, %	43	46	44	n/a	56	44
*DR4*, %	68	65	47	n/a	89	68
Protective HLA, %^b^	3	3	8	0	0	n/a
Antibodies^c^						
IAA positive, %	68	63	16	47	64	82
GADA positive, %	95	93	78	73	86	88
IA-2A positive, %	65	71	4	56	67	53
ZnT8A positive, %	62	66	2	n/a	67	63
Number of antibodies	3 [2, 3]; 2–4	3 [2, 3]; 2–4	1	n/a	3 [2, 4]; 2–4	3 [2, 4]; 2–4
OGTT glucose (mmol/l)						
0 min glucose	4.9 [4.6, 5.3]; 3.2–6.9	4.9 [4.6, 5.2]; 2.8–6.9	5 [4.8, 5.3]; 3.6–6.6	4.8 [4.4, 5.1]; 2.7–6.8	n/a	4.45 [3.9, 4.8]; 2.3–5.8
30 min glucose	8.3 [7.3, 9.3]; 2.5–13.4	8.3 [7.3, 9.4]; 4.6–13.4	8.1 [7.1, 9.0]; 3.9–14.7	7.9 [6.9, 9]; 3.8–14	n/a	7.6 [6.1, 8.7]; 3.3–11.8
60 min glucose	8 [6.7, 9.7]; 3.4–16.6	8.4 [6.9, 10]; 2.4–14.6	7.7 [6.2, 9.0]; 3.4–13.8	7.6 [5.9, 9]; 1.6–21.2	n/a	6.7 [5.7, 7.6]; 3.6–9.5
90 min glucose	7 [5.8, 8.4]; 3–14.3	7.4 [6.1, 9.1]; 2.8–13.9	6.6 [5.7, 7.9]; 2.9–13.2	6.6 [5.6, 7.9]; 2.1–17.9	n/a	5.9 [5.1, 6.4]; 3.9–9.9
120 min glucose	6.4 [5.6, 7.6]; 1.9–11.1	6.7 [5.7, 7.9]; 2.5–11.1	6.2 [5.3, 7.3]; 2.4–11.1	6.2 [5.3, 7.0]; 2.4–11.1	5.7 [4.9, 6.5]; 2.3–10.9	5.6 [4.9, 6.3]; 3.1–7.7
OGTT C-peptide (nmol/l)						
0 min C-peptide^d^	0.42 [0.29, 0.6]; 0.05–2.5	0.43 [0.29, 0.61]; 0.06–2.43	0.52 [0.36, 0.75]; 0.08–2.38	0.30 [0.22, 0.43]; 0.07–1.50	n/a	0.20 [0.15, 0.27]; 0.06–0.82
30 min C-peptide	1.52 [1.04, 2.16]; 0.06–10.6	1.41 [0.99, 2.03]; 0.07–7.69	1.90 [1.34, 2.56]; 0.24–7.14	1.17 [0.83, 1.60]; 0.20–4.23	n/a	0.79 [0.56, 1.08]; 0.16–1.79
60 min C-peptide	1.91 [1.31, 2.58]; 0.14–11.7	1.77 [1.25, 2.49]; 0.02–8.47	2.32 [1.71, 3.20]; 0.43–9.84	1.40 [1.00, 1.80]; 0.33–4.87	n/a	0.80 [0.63, 1.30]; 0.30–3.10
90 min C-peptide	1.8 [1.31, 2.5]; 0.18–7.77	1.77 [1.27, 2.51]; 0.48–10.67	2.21 [1.62, 2.99]; 0.33–8.73	1.37 [1.03, 1.83]; 0.30–4.60	n/a	n/a
120 min C-peptide^d^	1.73 [1.27, 2.44]; 0.26–9.86	1.68 [1.23, 2.41]; 0.20–10.13	2.07 [1.51, 2.81]; 0.23–9.03	1.30 [0.90, 1.70]; 0.10–4.07	1.11 [0.87, 1.41]; 0.01–3.14	0.77 [0.58, 1.07]; 0.15–3.40
Risk scores						
DPTRS	6.6 [5.8, 7.2]; 1.6–9.8	6.8 [6.1, 7.5]; 1.9–9.7	6.0 [5.1, 6.6]; 1.8–9.2	6.6 [6.0, 7.2]; 2.5–9.6	n/a	n/a
DPTRS60^d^	4.3 [3.6, 5.0]; −2.3–7.2	4.5 [3.7, 5.2]; 0.0–7.0	3.6 [2.6, 4.3]; −1.7–6.8	4.3 [3.8, 4.9]; 0.3–7.4	n/a	4.8 [4.3, 5.2]; 3.0–6.0
Index60^d^	0.4 [−0.3, 1.0]; −9.0–3.1	0.5 [−0.2, 1.2]; −5.6–2.9	−0.2 [−0.9, 0.5]; −6.3–2.5	0.6 [0.1, 1.1]; −2.7–4.4	n/a	0.1 [−0.2, 0.4]; −1.7–1.1
M_0_^d^	11.6 [11.1, 12.2]; 9.4–15.2	11.7 [11.1, 12.3]; 8.9–13.8	10.9 [10.5, 11.4]; 8.4–13.4	12 [11.4, 12.6]; 9.4–14.5	n/a	12.0 [11.5, 12.4]; 10.0–13.5
M_30_	11.6 [10.9, 12.3]; 6.5–15.4	11.8 [11.1, 12.6]; 7.6–14.6	10.8 [10.1, 11.4]; 7–13.9	12.1 [11.5, 12.8]; 9.0–15.7	n/a	12.3 [11.5, 12.8]; 9.6–14.2
M_60_	9.9 [9.1, 10.6]; 5.1–14.1	10.2 [9.4, 11.0]; 6.5–13.4	9.1 [8.4, 9.7]; 5–12.5	10.3 [9.7, 11.2]; 7.4–13.6	n/a	10.4 [9.9, 11.0]; 7.9–12.0
M_90_	10.1 [9.5, 10.8]; 7.1–14.3	10.4 [9.7, 11.1]; 7.3–13.7	9.4 [8.9, 9.9]; 7–13.4	10.4 [9.9, 11.2]; 7.8–14.8	n/a	n/a
M_120_^d^	10.9 [10.3, 11.5]; 7.9–14.9	11.1 [10.5, 11.9]; 7.7–14.8	10.2 [9.6, 10.8]; 7.2–14.3	11.3 [10.6, 12.0]; 8.3–15	11.0 [10.6, 11.7]; 9.0–14.9	11.3 [10.7, 11.7]; 9.2–12.9

Continuous data are median [Q1, Q3]; range

^a^Data available for only 808 and 601 participants in validation and single-antibody populations, respectively

^b^HLA-DQA1*01:02-DQB1*06:02 haplotype

^c^ZnT8 results available for only 211 participants in the TrialNet validation population

^d^Results available for only 79 of the 80 Fr1da participants

n/a, not available; T1D, type 1 diabetes

**Table 2:** Model performance in the TrialNet training and validation datasets

Population	Model	AUC (95% CI)	*p* v DPTRS	*p* v DPTRS60	*p* v Index60	*p* v AUC_max_	Sensitivity	Specificity	Accuracy
TrialNet training dataset	M_0_	0.689 (0.651, 0.728)	0.0019	0.0258	0.1905	<0.0001	0.710	0.552	0.583
	M_30_	0.735 (0.699, 0.771)	0.1306	0.8128	0.3828	0.0076	0.773	0.567	0.608
	M_60_	0.759 (0.723, 0.794)	0.9027	0.0955	0.0038	0.4626	0.798	0.573	0.618
	M_90_	0.763 (0.727, 0.799)	0.6550	0.0956	0.0069	n/a	0.803	0.574	0.619
	M_120_	0.748 (0.712, 0.785)	0.5301	0.5588	0.1191	0.0440	0.765	0.565	0.604
	DPTRS	0.757 (0.722, 0.792)	n/a	0.0041	0.0009	0.6550	0.790	0.571	0.614
	DPTRS60	0.739 (0.703, 0.775)	0.0041	n/a	0.0446	0.0956	0.761	0.564	0.603
	Index60	0.720 (0.683, 0.758)	0.0009	0.0446	n/a	0.0069	0.731	0.557	0.591
TrialNet validation dataset	M_0_	0.697 (0.661, 0.732)	0.0018	0.0603	0.1876	<0.0001	0.664	0.606	0.628
	M_30_	0.750 (0.717, 0.782)	0.4574	0.3631	0.1273	0.2509	0.732	0.650	0.682
	M_60_	0.760 (0.727, 0.793)	0.9916	0.0314	0.0031	0.9185	0.729	0.648	0.679
	M_90_	0.761 (0.728, 0.793)	0.9648	0.0641	0.0148	n/a	0.735	0.651	0.684
	M_120_	0.745 (0.712, 0.779)	0.2682	0.5330	0.2284	0.0361	0.708	0.634	0.663
	DPTRS	0.760 (0.727, 0.793)	n/a	0.0001	0.0009	0.9648	0.711	0.636	0.666
	DPTRS60	0.736 (0.701, 0.770)	0.0001	n/a	0.1992	0.0641	0.696	0.627	0.654
	Index60	0.725 (0.689, 0.760)	0.0009	0.1992	n/a	0.0148	0.708	0.634	0.663

Within each population, *p* v DPTRS, *p* v DPTRS60, *p* v Index60 and *p* v AUC_max_ are, respectively, *p* values for statistical comparisons with DPTRS, DPTRS60, Index60 and the model with the highest AUC, without correction for multiple comparisons

n/a, not applicable

**Fig. 1:**
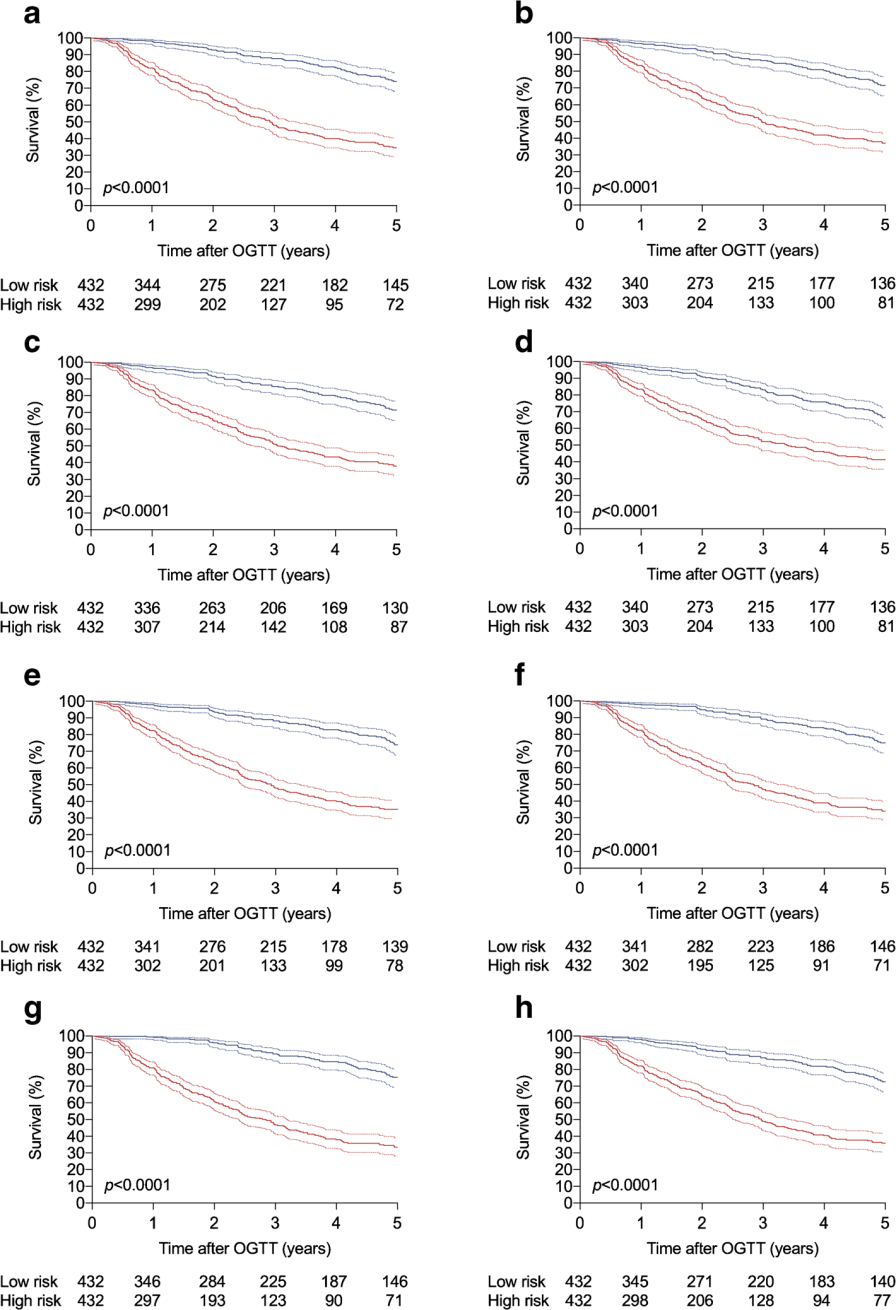
Survival curves in the TrialNet validation population. The percentage of participants free of progression to stage 3 type 1 diabetes with 95% CI is shown, stratified into high (red) and low (blue) risk according to the median value. (**a**–**h**) Risk scores calculated by the DPTRS, DPTRS60, Index60, M_0_, M_30_, M_60_, M_90_ and M_120_ models, respectively. The *p* values for curve comparisons are provided and numbers at risk are provided beneath each graph

Models were then validated using data from an independent TrialNet validation population of 864 participants (Table 1). Their median [Q1, Q3] duration of follow-up of 2.4 [1.0, 5.0] years after their first OGTT was significantly greater than the 1.8 [0.8, 3.2] years for the TrialNet training population (*p* < 0.0001), and the risk scores obtained from all models were significantly higher. The abilities of the single-time-point models to predict disease progression in individuals who scored above and below the median value were compared with those of the DPTRS, DPTRS60 and Index60 (Fig. 1). After 5 years of follow-up, M_30_, M_60_, M_90_ and M_120_ predicted stage 3 type 1 diabetes in approximately 25% of participants who scored below the median and 65% of participants who scored above it. When compared with the DPTRS, the M_30_, M_60_ and M_90_ models enabled slightly greater separation of high- and low-risk groups. Comparisons with DPTRS60 and Index60 showed that M_120_, as well as M_30_, M_60_ and M_90_, enabled greater separation. However, the AUCs for ROC curves for M_30_ (0.750), M_60_ (0.760), M_90_ (0.761) and M_120_ (0.745) did not differ significantly from the AUC of 0.760 for the DPTRS (Table 2). Measures of model sensitivity, specificity and accuracy are also presented in Table 2. The goodness of fit of each of the single-time-point models was confirmed using the Greenwood–D’Agostino–Nam calibration test.

We chose to focus analyses of additional at-risk populations on M_120_ because it performed well relative to DPTRS, DPTRS60 and Index60, and because it is most relevant for pre-symptomatic screening, where 120 min sampling is mandatory for type 1 diabetes staging, and therefore routinely performed for autoantibody-positive people. For completeness, the performance characteristics of all models in additional populations are presented in Table 3.

**Table 3:** Model performance in other populations

Population	Model	AUC (95% CI)	*p* v DPTRS	*p* v AUC_max_	Sensitivity	Specificity	Accuracy
TrialNet validation dataset, stage 1 T1D	M_0_	0.665 (0.614, 0.715)	0.7813	0.0334	0.671	0.566	0.595
	M_30_	0.698 (0.648, 0.747)	0.0835	n/a	0.729	0.587	0.626
	M_60_	0.682 (0.630, 0.734)	0.2214	0.2227	0.707	0.579	0.614
	M_90_	0.672 (0.621, 0.723)	0.4823	0.0865	0.693	0.574	0.606
	M_120_	0.651 (0.598, 0.704)	0.8405	0.0075	0.671	0.566	0.595
	DPTRS	0.656 (0.603, 0.709)	n/a	0.0835	0.636	0.552	0.575
	DPTRS60	0.651 (0.598, 0.704)	0.6032	0.0601	0.657	0.560	0.587
	Index60	0.636 (0.580, 0.692)	0.2385	0.0211	0.629	0.550	0.571
TrialNet validation dataset, stage 2 T1D	M_0_	0.663 (0.604, 0.721)	0.0007	0.0007	0.603	0.638	0.618
	M_30_	0.728 (0.675, 0.781)	0.0302	0.0302	0.638	0.684	0.658
	M_60_	0.764 (0.714, 0.814)	0.3681	0.3681	0.678	0.737	0.704
	M_90_	0.775 (0.727, 0.823)	0.7585	0.7585	0.658	0.711	0.681
	M_120_	0.753 (0.703, 0.803)	0.2344	0.2344	0.643	0.691	0.664
	DPTRS	0.781 (0.733, 0.830)	n/a	n/a	0.688	0.750	0.715
	DPTRS60	0.741 (0.689, 0.794)	<0.0001	<0.0001	0.673	0.730	0.698
	Index60	0.738 (0.686, 0.790)	0.0075	0.0075	0.663	0.717	0.687
TrialNet single-antibody dataset	M_0_	0.650 (0.549, 0.752)	0.0394	0.0006	0.727	0.513	0.525
	M_30_	0.760 (0.667, 0.853)	0.9761	0.1302	0.818	0.518	0.534
	M_60_	0.799 (0.708, 0.890)	0.0995	n/a	0.818	0.518	0.534
	M_90_	0.768 (0.668, 0.867)	0.7671	0.0588	0.848	0.520	0.538
	M_120_	0.701 (0.594, 0.808)	0.0448	0.0001	0.727	0.513	0.525
	DPTRS	0.761 (0.652, 0.870)	n/a	0.0995	0.758	0.515	0.528
	DPTRS60	0.752 (0.642, 0.862)	0.4518	0.0585	0.788	0.516	0.531
	Index60	0.784 (0.678, 0.889)	0.3086	0.4459	0.818	0.518	0.534
DPT-1	M_0_	0.611 (0.564, 0.658)	<0.0001	<0.0001	0.591	0.550	0.564
	M_30_	0.696 (0.653, 0.739)	<0.0001	<0.0001	0.683	0.598	0.627
	M_60_	0.741 (0.700, 0.781)	0.0009	0.0009	0.726	0.621	0.657
	M_90_	0.717 (0.675, 0.760)	<0.0001	<0.0001	0.697	0.606	0.637
	M_120_	0.694 (0.649, 0.738)	<0.0001	<0.0001	0.663	0.588	0.614
	DPTRS	0.800 (0.762, 0.838)	n/a	n/a	0.813	0.667	0.717
	DPTRS60	0.792 (0.754, 0.829)	0.2612	0.2612	0.798	0.659	0.707
	Index60	0.761 (0.720, 0.801)	0.0029	0.0029	0.745	0.631	0.671
TEDDY	M_120_	0.865 (0.792, 0.938)	n/a	n/a	0.909	0.580	0.632
Fr1da	M_0_	0.710 (0.549, 0.871)	n/a	0.7334	0.667	0.529	0.544
	M_30_	0.668 (0.510, 0.826)	n/a	0.2543	0.778	0.535	0.563
	M_60_	0.615 (0.438, 0.793)	n/a	0.0338	0.667	0.521	0.538
	M_120_	0.742 (0.596, 0.889)	n/a	n/a	0.889	0.557	0.595
	DPTRS60	0.567 (0.340, 0.794)	n/a	0.0019	0.333	0.486	0.468
	Index60	0.638 (0.440, 0.835)	n/a	0.0006	0.333	0.486	0.468

Within each population, *p* v DPTRS and *p* v AUC_max_ are, respectively, *p* values for statistical comparisons with DPTRS and the model with the highest AUC, without correction for multiple comparisons

n/a, not applicable; T1D, type 1 diabetes

**Fig. 2:**
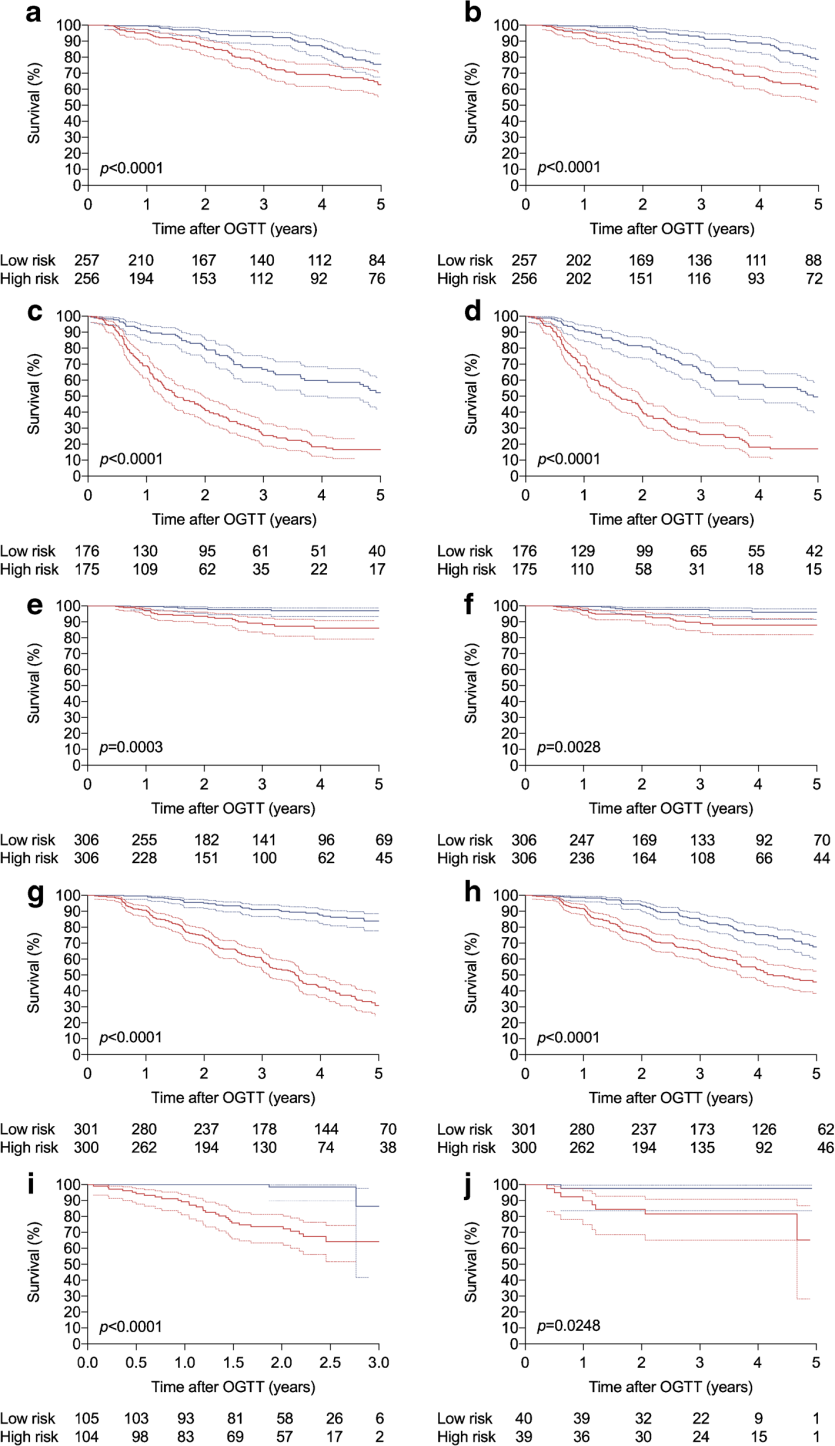
DPTRS and M_120_ survival curves in TrialNet sub-populations, DPT-1, TEDDY and Fr1da. Survival curves show the percentage of participants free of progression to stage 3 type 1 diabetes with 95% CI predicted by DPTRS (**a**, **c**, **e**, **g**) and M_120_ (**b**, **d**, **f**, **h**, **i**, **j**). Populations shown are the stage 1 (**a**, **b**) and stage 2 (**c**, **d**) subgroups of the TrialNet validation population, the TrialNet single-antibody population (**e**, **f**), DPT-1 (**g**, **h**), TEDDY (**i**) and Fr1da (**j**). Stratification into high (red) and low (blue) risk groups was according to the median value in each population. The *p* values for curve comparisons are provided and numbers at risk are provided beneath each graph

M_120_ reliably stratified stage 1 (normal glucose tolerance and HbA_1c_; *n* = 513) and stage 2 (fasting glucose 5.6 to 7.0 mmol/l; 30, 60 or 90 min glucose >11.1 mmol/l; 120 min glucose 7.8 to 11.1 mmol/l; and/or HbA_1c_ 39 to 46 mmol/mol; *n* = 351) subgroups of the TrialNet validation population, to a degree comparable to that of the DPTRS (Fig. 2a–d, Table 3).

M_120_ was then tested for its ability to stratify 612 TrialNet participants who tested positive to only a single autoantibody and were therefore not formally diagnosed with pre-symptomatic type 1 diabetes, which requires two or more autoantibody specificities [11]. This group included 89 participants (15%) who later developed multiple antibodies in TrialNet and whose data from a subsequent visit contributed to the TrialNet training (*n* = 72) or validation (*n* = 17) datasets. When compared with the two multiple-antibody datasets, the TrialNet single-antibody population had a lower rate of disease progression and lower risk scores (Table 1). Nonetheless, M_120_ could stratify this population into high- and low-risk subgroups, albeit less effectively than the DPTRS (Fig. 2e, f). The AUC of M_60_ in this population exceeded the AUCs of DPTRS, DPTRS60 and Index60, but the differences were not statistically significant (Table 3).

DPT-1 data were obtained from 601 relatives [21, 22]. When compared with the TrialNet training and validation populations, the earlier DPT-1 population had a higher age and HbA_1c_; lower incidence of IAA, GADA and IA-2A; and a lower C-peptide at all OGTT time points (Table 1). When DPT-1 was stratified by the median value into high- and low-risk groups, M_120_ enabled significant separation, with 32% of low-risk and 54% of high-risk participants progressing to stage 3 type 1 diabetes after 5 years (Fig. 2h). This degree of separation was lower than was observed in the TrialNet validation population (Fig. 1g), and also lower than could be achieved by the DPTRS, which predicted disease progression in 15% of low-risk and 70% of high-risk participants (Fig. 2f). The DPTRS AUC was significantly greater than the AUC of any other single-time-point model (Table 3).

The TEDDY study screened newborn, genetically at-risk children for islet autoantibodies every 3 to 6 months from age 3 months to 15 years. Multiple-autoantibody-positive children (*N* = 209; Table 1) underwent limited OGTT, wherein a single venous sample for glucose and C-peptide was collected 120 min after glucose challenge. In this group, M_120_ reliably predicted progression to stage 3 disease using the median value of 11.0 as the risk threshold (Fig. 2i), and its AUC was 0.865 (Table 3).

Finally, models were tested with data from 80 multiple-autoantibody-positive children participating in the Bavarian Fr1da general population screening programme [7] (Table 1). These children underwent an OGTT with sampling every 30 min. However, because C-peptide was not measured at 90 min, neither DPTRS nor M_90_ could be calculated. Based on the median score of 11.3 (Table 1), M_120_ significantly stratified the relatively small population of Fr1da participants (Fig. 2j). The M_120_ AUC of 0.742 was significantly greater than the AUCs for M_60_ (0.615), DPTRS60 (0.567) and Index60 (0.638) (Table 3).

## Discussion

We describe models to predict progression to insulin-dependent type 1 diabetes that are simpler than the previously validated DPTRS, DPTRS60 and Index60 risk scores and yet have comparable performance in the contemporary TrialNet and Fr1da populations. The models incorporated sex, age, BMI, HbA_1c_ and IA-2A status in combinations with glucose and C-peptide measures that are the basis of the DPTRS, DPTRS60 and Index60. In contrast to DPTRS, DPTRS60 and Index60, the new models focus on a single-time-point blood sample during the OGTT, decreasing the cost associated with analyte measurement. In addition, they do not require venous cannulation, which adds complexity and discomfort, particularly in young children. M_60_ and M_90_ were the most accurate single-time-point models in TrialNet. However, M_120_, based on a 120 min blood sample that is routinely used to stage type 1 diabetes, performed well in all populations other than DPT-1 and might therefore be best suited to current screening programmes of at-risk relatives [6] and, potentially, the general population [7, 8].

The universal use of sex, HbA_1c_ and IA-2A status by all single-time-point models suggests that their incorporation into DPTRS and DPTRS60 might improve the performance of these models, and that the performance of Index60 could be augmented by these measures together with age and BMI. Age, HbA_1c_ and IA-2A status are recognised risk factors for disease progression [7, 14, 28–30], and sex and BMI have been associated with progression to stage 3 diabetes in some [16, 30, 31], but not all [29], studies of autoantibody-positive people. Notably, although *HLA-DR3* and *-DR4* alleles have been described as predictors of progression from stage 1 to stage 3 disease in the TEDDY study [31], neither contributed to model performance.

Type 1 diabetes disease staging was introduced to educate the medical and lay communities about pre-symptomatic type 1 diabetes and the potential for its prevention using immune therapy [11]. Disease stages 1 and 2 also help classify the risk of progression to insulin dependence and have been used to define eligibility for prevention trials. Because disease staging requires a 120 min sample, M_120_ could be readily incorporated into current clinical workflows, thereby helping to improve clinical trial efficiency and, potentially, the identification of autoantibody-positive individuals at greatest risk of ketoacidosis, for whom education about symptoms of hyperglycaemia and close follow-up should be provided. It could also be used to identify individuals approaching insulin dependence who are currently best suited to receive immune therapy [4] as well as to identify high-risk single-antibody-positive individuals who would not currently meet entry criteria for TrialNet prevention trials. The key disadvantage of M_120_ compared with other single-time-point models is the time required to do the test, but perhaps this could be shortened if participants were provided with a kit that enabled them to record home fasting capillary blood glucose (for the purposes of type 1 diabetes staging), ingest glucose and then time their arrival to a collection centre for a single blood draw 120 min later.

In the TrialNet populations studied, the AUCs for the M_60_ and M_90_ models were greater than the AUCs of the other single-time-point models, and M_60_ performed better than M_0_, M_30_, M_90_ and M_120_ in DPT-1. These findings accord with a TrialNet study which demonstrated that a biphasic oral glucose response with a nadir at 60 or 90 min was associated with a low risk of disease progression [32], and the analysis of a combined DPT-1 and TrialNet dataset that formed the basis of the Index60 score, which identified 60 min measures of glucose and C-peptide as the strongest univariate predictors of stage 3 disease [17]. Overall, this suggests M_60_ could be used instead of DPTRS in TrialNet to simplify and decrease the cost of risk assessment, particularly if the 60 min glucose were used to diagnose diabetes mellitus, as suggested by other TrialNet studies [17, 18]. However, there has been a long-standing requirement for a 120 min glucose to diagnose diabetes mellitus [12], which is likely to endure. Furthermore, the very high M_120_ AUC of 0.865 in TEDDY and the superior performance of M_120_ relative to other models in Fr1da suggest that 120 min sampling might be optimal for disease staging and risk stratification outside of TrialNet.

When tested in DPT-1, the models developed in this study were less accurate than DPTRS, DPTRS60 and Index60. This discrepancy is at least in part because these three multiple-time-point models were developed using DPT-1 data [16–18], which differed from TrialNet with respect to age, autoantibody prevalence, HbA_1c_ and C-peptide. These population differences might be explained by the requirement in DPT-1 for participants to screen positive for islet cell antibodies by indirect immunofluorescence, the use of different autoantibody and C-peptide assays in DPT-1 that at times were performed many years after sample collection and, potentially, by changes in the contribution of environment to disease risk since the start of DPT-1 [33].

Several caveats should be mentioned. First, because TrialNet, TEDDY and Fr1da enrolled mostly individuals of European descent and used similar laboratory methods to measure C-peptide and HbA_1c_, the performance of the models in different contexts remains unproven. In addition, the Fr1da population was relatively small and therefore under-powered to assess the validity of all of the models, and thus further testing will be needed to confirm the utility of M_120_ in a general population setting. Finally, the thresholds used to define high and low risk for survival analyses were based on median values, which may not be optimal for specific populations. This being said, the DPTRS threshold of 6.8 used to define risk of progression to stage 3 type 1 diabetes in the TrialNet validation population was between the previously recommended thresholds of 6.5 for low risk [34] and 7.0 to 7.5 for high risk [34, 35]. Therefore, the M_120_ score of 11.1 used to stratify the TrialNet validation population would appear to be a reasonable threshold to apply to future TrialNet participants with stage 1 or 2 type 1 diabetes.

In summary, unbiased selection methods were applied to TrialNet data to develop equations to predict disease progression in pre-symptomatic type 1 diabetes. The M_120_ model, based on a single blood draw at 120 min of the OGTT, was identified as a comparably accurate yet more practical tool than the DPTRS, DPTRS60 or Index60. Its validity in different at-risk populations and its operational simplicity make M_120_ broadly applicable to current screening programmes and, potentially, for more routine clinical use with the advent of disease-modifying therapies.

## Supplementary information

ESM (PDF 90 kb)

## Data Availability

Data for TrialNet, TEDDY and DPT-1 can be obtained from the NIDDK data repository (https://repository.niddk.nih.gov/home/). Fr1da data are available upon reasonable request from A.-G. Ziegler, Institute of Diabetes Research, Helmholtz Zentrum München, German Research Center for Environmental Health, Munich-Neuherberg, Germany.

## Abbreviations

AIC: Akaike’s information criterion
DPT-1: Diabetes Prevention Trial–Type 1
DPTRS: Diabetes Prevention Trial Risk Score
GADA: GAD autoantibodies
IA-2A: Insulinoma antigen-2 autoantibodies
IAA: Insulin autoantibodies
ROC: Receiver operating characteristic
TEDDY: The Environmental Determinants of Diabetes in the Young
ZnT8A: Zinc transporter-8 autoantibodies
