# Supplementary material for: Simplifying prediction of disease progression in pre-symptomatic type 1 diabetes using a single blood sample
Source: Diabetologia. 2021 Aug 2;64(11):2432–44. doi: 10.1007/s00125-021-05523-2 (PMC8494707; doi:10.1007/s00125-021-05523-2)
Supplement: Supplementary file 1 — (PDF 90 kb) [file 125_2021_5523_MOESM1_ESM.pdf]

ESM Table 1. Inputs used to train the models.

Input	***Notes***
AGE	Age in years at index OGTT
SEX	male or female
BMI	in kg/m^2
HBA1C	HbA1c in Per centage units
IAA	Insulin antibody (positive or negative)
GADA	GAD antibody (positive or negative)
IA2A	IA2 antibody (positive or negative)
ZNT8A	ZnT8 antibody (positive or negative)
ABNO	Number of antibody specificities
Antibody profile	
GI	GADA and IAA only (yes or no)
GIZ	GADA, IAA and ZnT8A only (yes or no)
GA	GADA and IA2A only (yes or no)
GAZ	GADA, IA2A and ZnT8A only (yes or no)
GAIZ	All 4 antibodies (yes or no)
OTHER	Other antibody combinations (yes or no)
HLA results	
PROTECTIVE HLA	Absence/Presence of DQA1*0102, DQB1*0602
HLA-DR3	Absence/Presence DRB*0301 OR 0305, DQA*0501, DQB*0201
HLA-DR4	Absence/Presence DRB*0401, 0402, 0403, etc. DQA*0301 DQB*0302
OGTT measurements	
GLUC 0	Fasting glucose (mg/dL)
GLUC 30	30-minute glucose (mg/dL)
GLUC 60	60-minute glucose (mg/dL)
GLUC 90	90-minute glucose (mg/dL)
GLUC 120	120-minute glucose (mg/dL)
CPEP 0	Fasting C-peptide (ng/mL)
CPEP 30	30-minute C-peptide (ng/mL)
CPEP 60	60-minute C-peptide (ng/mL)
CPEP 90	90-minute C-peptide (ng/mL)
CPEP 120	120-minute C-peptide (ng/mL)

ESM Table 2. Models to predict disease progression

		M_0	M_{30}	M_{60}	M_{90}	M_{120}
AIC		2838.5	2764.9	2722.8	2723.2	2731.9
Sex (M=1, F=2)	β	0.346	0.419	0.434	0.499	0.448
	se(β)	0.133	0.132	0.133	0.134	0.134
	|β|/se(β)	2.60	3.17	3.26	3.72	3.34
Presence of IA2 (N=0, Y=1)	β	0.832	0.698	0.645	0.624	0.631
	se(β)	0.163	0.164	0.164	0.165	0.165
	|β|/se(β)	5.10	4.26	3.93	3.78	3.82
Age (years)	β	-0.0306	-0.0387	-0.0330	-0.0319	-0.0302
	se(β)	0.0096	0.0109	0.0107	0.0107	0.0107
	|β|/se(β)	3.19	3.55	3.08	2.98	2.82
BMI (kg/m^2)	β	-	0.0858	0.0744	0.0603	0.0605
	se(β)	-	0.0198	0.0186	0.0189	0.0193
	|β|/se(β)	-	4.33	4.00	3.19	3.13
HbA1c (%)	β	1.846	1.603	1.389	1.344	1.380
	se(β)	0.228	0.218	0.22	0.22	0.225
	|β|/se(β)	8.10	7.35	6.31	6.11	6.13
Glucose 0min (mg/dL)	β	0.0203	-	-	-	-
	se(β)	0.00836	-	-	-	-
	|β|/se(β)	2.43	-	-	-	-
C-peptide 0min (ng/mL)	β	-0.184	-	-	-	-
	se(β)	0.109	-	-	-	-
	|β|/se(β)	1.69	-	-	-	-
Glucose 30min (mg/dL)	β	-	0.0173	-	-	-
	se(β)	-	0.0026	-	-	-
	|β|/se(β)	-	6.65	-	-	-
C-peptide 30min (ng/mL)	β	-	-0.283	-	-	-
	se(β)	-	0.038	-	-	-
	|β|/se(β)	-	7.45	-	-	-
Glucose 60min (mg/dL)	β	-	-	0.0152	-	-
	se(β)	-	-	0.0017	-	-
	|β|/se(β)	-	-	8.94	-	-
C-peptide 60min (ng/mL)	β	-	-	-0.240	-	-
	se(β)	-	-	0.034	-	-
	|β|/se(β)	-	-	7.06	-	-
Glucose 90min (mg/dL)	β	-	-	-	0.0187	-
	se(β)	-	-	-	0.0017	-
	|β|/se(β)	-	-	-	11.00	-
C-peptide 90min (ng/mL)	β	-	-	-	-0.171	-
	se(β)	-	-	-	0.031	-
	|β|/se(β)	-	-	-	5.52	-
Glucose 120min (mg/dL)	β	-	-	-	-	0.0265
	se(β)	-	-	-	-	0.0025
	|β|/se(β)	-	-	-	-	10.60
C-peptide 120min (ng/mL)	β	-	-	-	-	-0.191
	se(β)	-	-	-	-	0.035
	|β|/se(β)	-	-	-	-	5.46

AIC: Akaike information criterion in the TrialNet training dataset (lower values correspond to greater accuracy).

Each risk score is the sum of the products of each input and its β coefficient. For example, M_0= Sex x 0.346 + IA-2A x 0.163 + Age x -0.0306 + HbA1c x 1.846 + $Gluc_{0min}$ x 0.0203 + $CPep_{0min}$ x -0.184
